# The role of plasma triglyceride/high-density lipoprotein cholesterol ratio to predict cardiovascular outcomes in chronic kidney disease

**DOI:** 10.1186/s12944-015-0031-4

**Published:** 2015-04-16

**Authors:** Alper Sonmez, Mahmut Ilker Yilmaz, Mutlu Saglam, Hilmi Umut Unal, Mahmut Gok, Hakki Cetinkaya, Murat Karaman, Cem Haymana, Tayfun Eyileten, Yusuf Oguz, Abdulgaffar Vural, Manfredi Rizzo, Peter P Toth

**Affiliations:** Department of Endocrinology and Metabolism, Gulhane School of Medicine, 06018 Etlik, Ankara Turkey; Department of Nephrology, Gulhane School of Medicine, 06018 Etlik, Ankara Turkey; Department of Radiology, Gulhane School of Medicine, 06018 Etlik, Ankara Turkey; Biomedical Department of Internal Medicine and Medical Specialties, University of Palermo, Palermo, Italy; University of Illinois School of Medicine, Peoria, Il USA

**Keywords:** Asymmetric dimethyl arginine, Chronic kidney disease, Flow mediated dilatation, Triglyceride to HDL-cholesterol ratio

## Abstract

**Background:**

Cardiovascular disease (CVD) risk is substantially increased in subjects with chronic kidney disease (CKD). The Triglycerides (TG) to High-Density Lipoprotein Cholesterol (HDL-C) ratio is an indirect measure of insulin resistance and an independent predictor of cardiovascular risk. No study to date has been performed to evaluate whether the TG/HDL-C ratio predicts CVD risk in patients with CKD.

**Methods:**

A total of 197 patients (age 53 ± 12 years) with CKD Stages 1 to 5, were enrolled in this longitudinal, observational, retrospective study. TG/HDL-C ratio, HOMA-IR indexes, serum asymmetric dimethyl arginine (ADMA), high sensitivity C-reactive protein (CRP), parathyroid hormone (PTH), calcium, phosphorous, estimated glomerular filtration rate (eGFR), and albumin levels were measured. Flow mediated vasodilatation (FMD) of the brachial artery was assessed by using high-resolution ultrasonography.

**Results:**

A total of 11 cardiovascular (CV) deaths and 43 nonfatal CV events were registered in a mean follow-up period of 30 (range 9 to 35) months. Subjects with TG/HDL-C ratios above the median values (>3.29) had significantly higher plasma ADMA, PTH, and phosphorous levels (p = 0.04, p = 0.02, p = 0.01 respectively) and lower eGFR and FMD values (p = 0.03, p < 0.001 respectively). The TG/HDL-C ratio was an independent determinant of FMD (β = −0.25 p = 0.02) along with TG, HDL-C, hsCRP, serum albumin, phosphate levels, systolic blood pressure, PTH, eGFR and the presence of diabetes mellitus. The TG/HDL-C ratio was also a significant independent determinant of cardiovascular outcomes [HR: 1.36 (1.11-1.67) (p = 0.003)] along with plasma ADMA levels [HR: 1.31 (1.13-1.52) (p < 0.001)] and a history of diabetes mellitus [HR: 4.82 (2.80-8.37) (p < 0.001)].

**Conclusion:**

This study demonstrates that the elevated TG/HDL-C ratio predicts poor CVD outcome in subjects with CKD. Being a simple, inexpensive, and reproducible marker of CVD risk, the TG/HDL-C ratio may emerge as a novel and reliable indicator among the many well-established markers of CVD risk in CKD.

**Systematic review registration:**

Clinical trial registration number and date: NCT02113462 / 10-04-2014.

## Background

Chronic kidney disease (CKD) is a major risk factor for premature cardiovascular diseases (CVD) and mortality [1] and a global public health problem [2]. Patients with CKD frequently die from CVD before progressing to end-stage renal disease (ESRD) [3]. The reasons for the elevated risk of CVD in patients with CKD are not fully elucidated. The classical Framingham risk factors do not fully account for the increased CVD risk and other factors such as inflammation, oxidative stress, insulin resistance and endothelial dysfunction are among the major contributors to the increased CVD risk associated with CKD [4-6]. However, none of the biomarkers of inflammation, oxidative stress, endothelial dysfunction, vascular calcification or insulin resistance are good enough to be used as prognostic tools [7]. A simple, widely available, relatively inexpensive, and generally reproducible marker to predict the CVD risk in subjects with CKD is needed.

The ratio of triglycerides (TG) to High Density Lipoprotein Cholesterol (HDL-C) is easily measured. Elevated TG/HDL-C ratio it is an acknowledged marker of insulin resistance and it is an independent predictor of cardiovascular risk [8-11]. The role of TG/HDL-C ratio in predicting CVD risk has been tested in several metabolic disorders such as diabetes mellitus, hypertension, and nonalcoholic fatty liver disease (NAFLD) [12-15]. To our knowledge no study has been performed to estimate the role of TG/HDL-C ratio in predicting CVD risk in patients with CKD.

This study is designed to evaluate the relationship between TG/HDL-C ratio and endothelial functions in patients with CKD and to validate the role of TG/HDL-C ratio in predicting CVD outcomes in these patients. In order to comprehensively test if the TG/HDL-C ratio is an independent predictor of CVD events, we adjusted for several well-established risk factor covariates, including flow mediated dilatation (FMD), asymmetric dimethyl arginine (ADMA), high sensitivity CRP, homeostasis model of assessment (HOMA-IR), parathyroid hormone, serum calcium, phosphorous and albumin levels in the COX regression model.

## Methods

### Patients and study design

The ethical committee of Gulhane School of Medicine (Etlik-Ankara, Turkey) approved the study, and informed consent was obtained from each patient. Between January 2009 and January 2013, 644 patients were referred to the Renal Unit of the Gulhane School of Medicine Medical Center, Ankara, Turkey, because of suspected or manifest CKD. All patients were diagnosed as having CKD according to their estimated glomerular filtration rate (eGFR) and/or the evidence of kidney disease such as proteinuria or hematuria; a genetic diagnosis of kidney disease (e.g. polycystic kidney disease); or evidence of structurally abnormal kidneys, as defined by the National Kidney Foundation K/DQOI Guidelines [16]. By protocol, and in order to minimize any confounding effects of conditions that may influence endothelial dysfunction, 447 patients who were taking drugs that may influence endothelial function were excluded, including angiotensin converting enzyme inhibitors (ACEIs; n = 111), angiotensin receptor blockers (ARBs; n = 99), statins (n = 65), EPO (n = 18) or supplemental vitamin pills (n = 13). Otherwise, other exclusion criteria including acute infections, and unwillingness to participate in the study were applied (n = 40). One hundred and one eligible patients dropped out for the following reasons: lost to follow-up or transferred to other dialysis units (n = 73), viral hepatitis (n = 5), vasculitis (n = 12), or withdrew consent (n = 11). As a result, 197 patients with a mean age of 53 ± 12 years were included in the study.

The characteristics of CKD subjects are given in Table 1. Arterial blood pressures were measured by a physician in the morning three consecutive times after a 15-min resting period, and mean values were calculated for systolic and diastolic pressure in all patients. Hypertension was defined as systolic blood pressure (SBP) ≥140 mmHg or diastolic blood pressure (DBP) ≥ 90 mmHg on repeated measurements, or the use of antihypertensive drugs. Thirty-three of the patients were on antihypertensive medications other than the renin angiotensin aldosterone system blockers (17 patients were treated with calcium channel antagonists, 5 with beta-blocker agents, 8 with alpha blockers and 3 with loop diuretics). At time of evaluation, forty-three of the patients were on anti-diabetic therapy (13 patients were treated with oral anti-diabetic agents and 30 with insulin). As soon as diabetic nephropathy was diagnosed, all patients taking oral anti-diabetic medications were transitioned to insulin. Forty six of these patients (23%) had a history of CVD as defined by medical history and/or clinical findings at time of enrollment. Of these 46 patients, 4 had suffered from cerebrovascular accidents (stroke), 36 from cardiovascular disease (acute myocardial infarction, angina pectoris or had undergone coronary artery bypass surgery or percutaneous angioplasty); 6 had a history of peripheral ischemic atherosclerotic vascular disease and 1 patient had a history of an aortic aneurysm. Smoking habits were recorded as follows: whereas 87 patients were former or current smokers, 110 were non-smokers. Patients’ CKD severity were classified with respect to eGFR levels from stage 1 to 5 as determined by K/DOQI, which was calculated according to the simplified version of the Modification of Diet, in Renal Disease (MDRD) formula as defined by Levy et al. [17] [GFR = 186 x Pcr-1.154 x age-0.203 x 1.212 (if black) x 0.742 (if female)]. Additionally, patients were followed for time-to-event analysis of cardiovascular outcomes, any non-fatal cardiovascular event (stroke, myocardial infarction, complications of PVD and aneurysm rupture) or death, whichever came first. Cardiovascular events were assessed/adjudicated during follow-up visits. If patients failed to follow-up, events were interrogated/established via telephone interview.

**Table 1:** **Demographic and clinical characteristics of the study patients as stratified to CKD stages**

**eGFR (ml/min/1.73 m** ^**2**^ **)**	**≥90**	**60-89**	**30-59**	**15-29**	**<15**
	**Stage 1**	**Stage 2**	**Stage 3**	**Stage 4**	**Stage 5**
	**(n = 39)**	**(n = 40)**	**(n = 39)**	**(n = 37)**	**(n = 42)**
**Age (years)**	52 (30–73)	55 (32–71)	53 (31–73)	56 (33–73)	51 (30–73)
**Sex (M/F)**	20/19	19/21	19/20	20/17	21/21
**BMI (kg/m** ^**2**^ **)**	26.4 ± 2.3	26.8 ± 3.3	25.6 ± 2.8	26.2 ± 2.9	25.2 ± 2.6
**History of CVD (n)**					
**Fatal and nonfatal MI, acute coronary syndrome**	5	6	8	10	7
**Stroke**	0	-	3	-	1
**Peripheral vascular disease**	1	1	1	2	1
**Aortic aneurysm**	-	-	-	-	1
**Etiology of CKD (n)**					
**Diabetes**	6	10	7	12	8
**Glomerulonephritis**	6	7	7	5	6
**Hypertension**	5	8	7	6	7
**Polycystic kidney disease**	2	2	3	3	2
**Reflux Nephropathy**	2	1	2	1	2
**Unknown**	18	12	13	10	17
**Smoking, current or former (n)**	22	13	14	21	17
**CV events (n)**	7	10	8	13	16

### Laboratory measurements

All samples were obtained in the morning after 12 hours of fasting, for measurement of fasting plasma glucose (FPG), serum albumin, total serum cholesterol (TC), TG, HDL-C, low-density lipoprotein (LDL) cholesterol. TC TG and HDL-C were measured by enzymatic colorimetric method with Olympus AU 600 auto analyzer using reagents from Olympus Diagnostics, GmbH (Hamburg, Germany). LDL cholesterol was calculated by Friedewald’s formula [18]. For the measurement of hsCRP, serum samples were diluted with a ratio of 1/101 with the diluents solution. Calibrators, kit controls and serum samples were all added on each micro well with an incubation period of 30 minutes. After 3 washing intervals 100 μL enzyme conjugate (peroxidase labeled anti-CRP) was added on each micro well for additional 15 minutes incubation in room temperature in dark. The reaction was stopped with a stop solution and photometric measurement was performed at the 450 nm wavelength. The serum concentration was calculated as mg/l with a graphic that was made by noting the absorbance levels of the calibrators. The serum basal insulin value was determined by the coated tube method (DPC-USA). Insulin resistance score, HOMA-IR, was computed by the following formula [19]: HOMA-IR = FPG (mg/dL) x Immunoreactive insulin (IRI) (μIU/mL)/405.

#### Measurement of ADMA

ADMA is an endogenous inhibitor of the nitric oxide production, a key chemical involved in normal endothelial function and cardiovascular health. Measurement of serum ADMA was done using high performance liquid chromatography, as described by Chen et al. [20]. In brief, to 1 ml serum, 20 mg of 5-sulfosalisilic acid (5-SSA) was added and the mixture was left in an ice-bath for 10 min. The precipitated protein was removed by centrifugation at 2000 g for 10 min. Ten micro liters of the supernatant which was filtered through a 0.2 μm filter was mixed with 100 μl of derivatization reagent (prepared by dissolving 10 mg o-phtaldialdehyde in 0.5 ml of methanol, 2 ml of 0.4 M borate buffer (pH 10.0) and 30 μl of 2-mercaptoethanol) and then injected into the chromatographic system. Separation of ADMA was achieved with a 150x4 mm I.D. Nova-pak C18 column with a particle size of 5 μm (Waters, Millipore, Milford, MA, USA) using 50 mM sodium acetate (pH 6.8), methanol and tetrahydrofurane as mobile phase (A, 82:17:1; B, 22:77:1) at a flow-rate of 1.0 ml/min. The areas of peaks detected by the fluorescent detector (Ex: 338 nm; Em: 425 nm) were used as quantification. The variability of the method was less than 7%, and the detection limit of the assay was 0.01 μM.

### Vasoreactivity assessment

Flow mediated dilatation reflects the NO bioavailability to the endothelium and is well determined by the serum ADMA concentration [21]. Both FMD and endothelium-independent vasodilatation (NMD) of the brachial artery were assessed non-invasively, using high resolution ultrasonography as described by Celermajer et al. [22]. The method for the vascular assessment met the criteria which were mentioned by the International Brachial Artery Reactivity Task Force [23]. Measurements were made by a single observer using an ATL 5000 ultrasound system (Advanced Technology Laboratories Inc., Bothell, WA., USA) with a 12-Mhz prob. The subjects remained at rest in the supine position for at least 15 min before the examination started. The arm of a given patient was comfortably immobilized in the extended position to allow consistent recording of the brachial artery 2–4 cm above the antecubital fossa. Three adjacent measurements of end-diastolic brachial artery diameter were made from single 2-D frames. All ultrasound images were recorded on S-VHS videotape for subsequent blinded analysis. A pneumatic tourniquet was inflated to 200 mmHg with obliteration of the radial pulse. After 5 minutes the cuff was deflated. Flow measurements were made 60s post-deflation. After a further 15 min, measurements were repeated and again 3 min after administration of sublingual glyceryl trinitrate 400 μg. The maximum FMD and NMD dilatation diameters were calculated as the average of the three consecutive maximum diameter measurements. The FMD and NMD were then calculated as the percent change in diameter compared with baseline resting diameters.

### Statistical analysis

All the statistical analyses were performed by using SPSS 17.0 (SPSS Inc., Chicago, IL) statistical package. Non-normally distributed variables were expressed as median (range) and normally distributed variables were as mean ± SD, as appropriate. A p value <0.05 was considered to be statistically significant. Between-group comparisons were assessed for nominal variables with the Chi-square test, and by Kruskal-Wallis test (ANOVA) for the remaining variables. Spearman’s rank correlation was used to determine correlations between paired variables. Stepwise multivariate regression analysis was used to assess the predictors for FMD levels. Survival and time-to-event analysis of cardiovascular outcomes (using a composite of fatal and non-fatal events) was done using Cox proportional hazards model, including adjustment for potential confounding factors.

We used a stepwise analysis in the multivariate COX regression. In the SPSS program, the multivariate COX analysis starts by inserting all the parameters which correlate with CV events in the univariate analysis. Covariates that do not contribute significantly to the adjusted models are then eliminated in stepwise fashion. Data is presented in the form of Hazard ratios (HR) and 95% confidence intervals (CI).

## Results

Cardiovascular outcomes were determined from the day of examination onwards, with a mean follow-up period of 30 (range 9 to 35) months. Thirteen patients died, 11 of which were due to cardiovascular causes, one due to malignancy, and one due to infection. Cardiovascular mortality (n = 11) was defined as death due to coronary heart disease (n = 7), sudden death (n = 1), stroke (n = 2) or complicated peripheral vascular disease (n = 1).

In addition to the 11 cardiovascular deaths, 43 non-fatal cardiovascular events were registered during the follow-up as follows: stroke (n = 14); myocardial infarction (n = 23); peripheral vascular disease (n = 4) and aortic aneurysm (n = 2).

The demographic and clinical characteristics of the study patients as stratified to CKD stages are given in Table 1. The demographic and laboratory parameters, stratified according to the median TG/HDL-C value (3.29), are given in Table 2. The CKD distribution was not significantly different according to the TG/HDL-C groups. According to the results, plasma ADMA, PTH and phosphorus levels were significantly higher (p = 0.04, p = 0.02, p = 0.01) and eGFR and FMD values were significantly lower (p = 0.03, p < 0.001) in subjects with the higher TG/HDL-C ratio.

**Table 2:** **The demographic and laboratory parameters of CKD patients above and below median TG/HDL-C ratio**

	**TG/** **HDL-C** **ratio**	**TG/** **HDL-C** **ratio**	*P value
	**<3.29**	**>3.29**	
**ADMA (μmol/l)**	2.7 ± 1.6	3.2 ± 1.7	**0.04**
**PTH (pg/ml)**	122.5 ± 88.4	154.2 ± 101.1	**0.02**
**eGFR (ml/min)**	56.7 ± 33.4	46.6 ± 31.7	**0.03**
**FMD (%)**	7.2 ± 1.3	6.5 ± 1.2	**<0.001**
**hsCRP (mg/l)**	14.6 ± 7.8	16.0 ± 8.1	0.21
**SBP (mmHg)**	133.3 ± 8.2	136.1 ± 11.9	0.06
**DBP (mmHg)**	84.2 ± 4.4	84.5 ± 5.1	0.57
**HOMA-IR**	1.7 ± 0.7	1.8 ± 0.7	0.90
**Age (years)**	52 ± 12	54 ± 12	0.08
**Gender (M/F)**	51/47	50/49	0.89
**BMI (kg/m** ^**2**^ **)**	26.1 ± 2.8	26.2 ± 2.8	0.88
**Total Cholesterol (mg/dl)**	191.3 ± 23.9	201.2 ± 23.5	**0.04**
**Triglyceride (mg/dl)**	131.3 ± 15.8	150.5 ± 18.3	**<0.001**
**LDL-cholesterol (mg/dl)**	125.9 ± 16.4	129.6 ± 21.6	0.18
**HDL-cholesterol (mg/dl)**	47.1 ± 6.9	37.3 ± 6.2	**<0.001**
**Triglyceride/HDL-C ratio**	2.8 ± 0.4	4.2 ± 1.1	**<0.001**
**Albumin (g/dl)**	3.9 ± 0.3	4.0 ± 0.4	0.29
**Calcium (mg/dl)**	8.5 ± 0.6	8.4 ± 0.5	0.29
**Phosphate (mg/dl)**	4.9 ± 1.4	5.6 ± 1.7	**0.01**
**NMD (%)**	12.9 ± 0.6	12.7 ± 0.8	0.71

*Student’s t test.

The correlates of endothelial function in the study group are investigated as the univariate and multivariate associates of FMD (Table 3). The TG/HDL-C ratio was an independent determinant of FMD (β = 0.25 p = 0.02) together with the TG, HDL-C, hsCRP, serum albumin, phosphate levels, SBP, PTH, eGFR and the presence of diabetes mellitus.

**Table 3:** **Univariate and multivariate associates of flow mediated dilatation (FMD) in non-dialysis CKD patients**

	**FMD**	
**Parameters**	**Univariate** ^**0**^ **ρ**	**Multivariate** ^**1**^ **β (P)**
**ADMA (μmol/l)**	**−0.58 (<0.001)**	NS
**Total Cholesterol (mg/dl)**	**−0.33 (<0.001)**	NS
**Triglyceride (mg/dl)**	**−0.19 (0.009)**	**−0.22 (0.001)**
**LDL-cholesterol (mg/dl)**	−0.14 (0.06)	NS
**HDL-cholesterol (mg/dl)**	**0.37 (<0.001)**	**0.32 (<0.001)**
**Triglyceride/HDL-C ratio**	**−0.34 (<0.001)**	**−0.25 (0.02)**
**Total cholesterol/HDL-C ratio**	**0.30 (<0.001)**	NS
**NMD (%)**	**0.45 (<0.001)**	**0.24 (<0.001)**
**Diabetes (yes/no)**	**−0.21 (0.004)**	**- 0.13 (0.001)**
**Previous CVD (yes/no)**	−0.07 (0.37)	NS
**hsCRP (mg/l)**	**−0.57 (<0.001)**	**−0.10 (0.03)**
**Smoking (yes/no)**	0.04 (0.45)	NS
**SBP (mmHg)**	**−0.23 (0.001)**	**−0.10 (0.01)**
**DBP (mmHg)**	**−0.20 (0.005)**	NS
**HOMA-IR**	**−0.19 (0.008)**	NS
**Age (yr)**	−0.05 (0.45)	NS
**Gender (M/F)**	0.03 (0.63)	NS
**S-albumin (g/dl)**	**0.26 (<0.001)**	**0.13 (0.02)**
**Serum calcium (mg/dl)**	**0.46 (<0.001)**	NS
**Serum phosphate (mg/dl)**	**−0.65 (<0.001)**	**−0.13 (0.009)**
**iPTH (pg/ml)**	**−0.60 (<0.001)**	**−0.41 (<0.001)**
**eGFR (ml/min)**	**0.80 (<0.001)**	**0.40 (<0.001)**

^**0**^Denoted is statistically significant (P < 0.05) ρ values as assessed by Spearman Rank’s test, as well as β estimate and P value from multivariate regression model. The r^2^ of the multivariate model was 0.76. Variables known to influence FMD levels (age, gender. Diabetes (yes/no), ADMA, Previous CVD, HOMA-IR, calcium, phosphate, SBP, DBP, hsCRP, NMD, albumin, iPTH, eGFR, TG/HDL-C ratio are inserted in the multivariate analyses.

The COX analysis was performed in order to establish which covariates were independent predictors of cardiovascular outcomes (Table 4). The TG/HDL-C ratio [HR: 1.36 (1.11-1.67) (p = 0.003)], the plasma ADMA levels [HR: 1.31 (1.13-1.52) (p < 0.001)] and the history of diabetes [HR: 4.82 (2.80-8.37) (p < 0.001)] are all significant independent predictors of cardiovascular outcomes in patients with CKD.

**Table 4:** **Univariate and multivariate COX analysis predicting for cardiovascular outcomes (a composite of 54 fatal and non-fatal events)**

	**Crude analysis**		**Terminal Model**	
**ADMA (μmol/l)**	1.36 (1.18-1.56)	<0.001	1.31 (1.13-1.52)	<0.001
**TG/HDL-C ratio**	1.56 (1.29-1.90)	<0.001	1.36 (1.11-1.67)	0.003
**History of Diabetes (Yes/No)**	4.91 (2.85-8.44)	<0.001	4.82 (2.80-8.37)	<0.001

Represented are Hazard Ratios (and 95% confidence intervals) in univariate (crude) Cox model and after systematic adjustments. Saturated model at first step include age (in years). Sex (women as reference). eGFR (in ml/min). hsCRP, FMD, calcium, phosphate, PTH, smoking, serum albumin, total cholesterol, triglyceride, LDL-cholesterol, HDL-cholesterol, SBP, DBP, HOMA-IR, ADMA, diabetes (absence as reference) and medical history of cardiovascular disease (absence as reference) at baseline.

FMD. Flow mediated dilatation; hsCRP. High sensitivity C reactive protein; eGFR. Estimated glomerular filtration rate.

**Figure 1:**
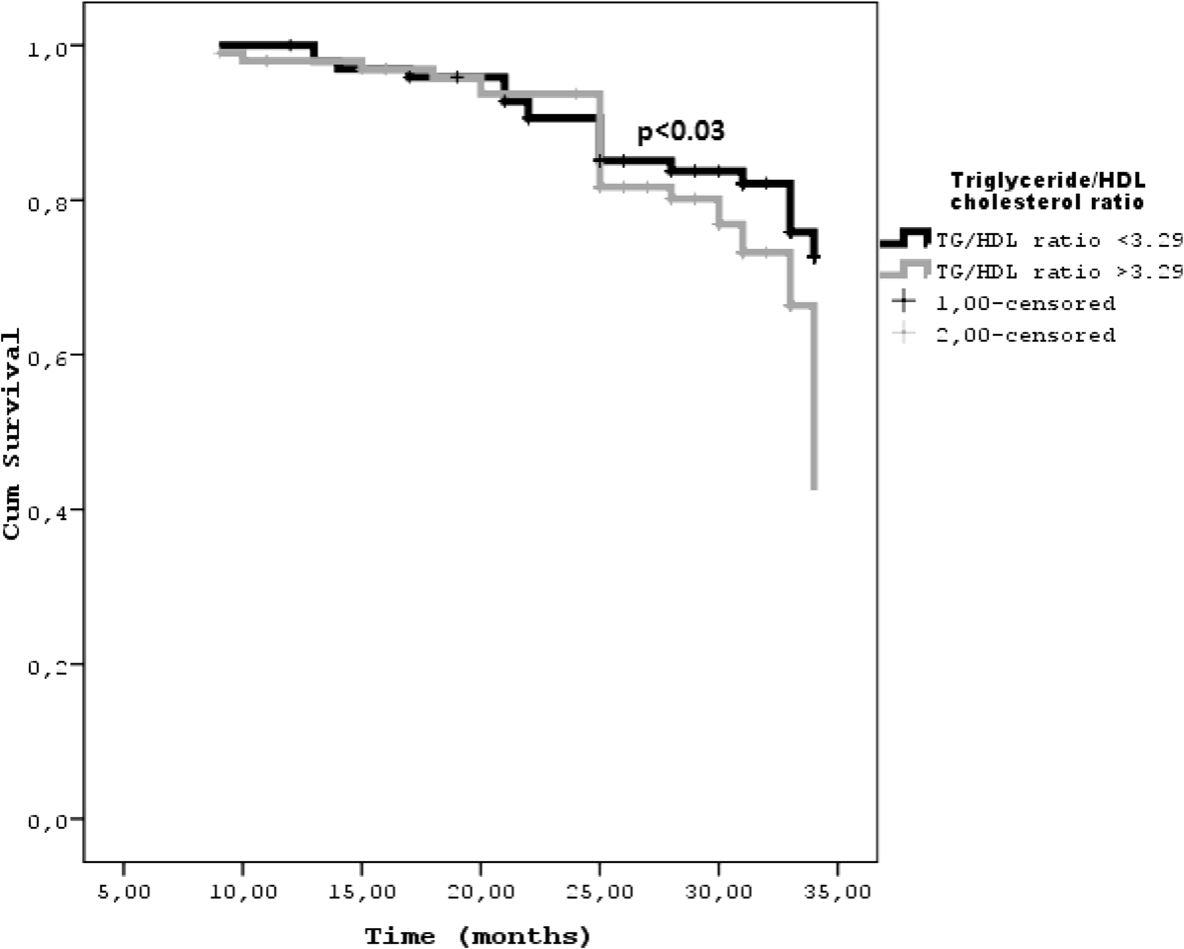
Kaplan-Meier survival curves according to TG/HDL ratio <3.29 or ≥3.29 at 30 months.

Kaplan Meier survival curves were generated to establish the impact of the TG/HDL-C ratio on the cumulative survival of the cohort (Figure 1). We further analyzed the predictive power of the median TG/HDL-C ratio in each CKD stage for all-cause mortality and cardiovascular events. In every CKD stage, a TG/HDL-C ratio above the median increases the risk for cardiovascular events (p < 0.03).

## Discussion

The results of the present study show that higher TG/HDL-C ratios in the CKD patients are associated with increased cardiovascular disease risk as shown by the elevated ADMA, PTH, phosphate and lower FMD and eGFR levels. Also, the TG/HDL-C ratio appears to be a significant determinant of the endothelial dysfunction and a simple predictor of cardiovascular outcomes along with the history of diabetes and high ADMA levels. The implications of these findings are examined below.

Patients with CKD have significantly increased cardiovascular risk as they mostly die from CVD even before the implementation of renal replacement therapy [1-3]. Neither the classical risk factors nor the novel ones fully account for the increased risk of cardiac mortality in CKD [4-7,24,25]. Likewise, although dyslipidemia is a common feature of CKD, traditional lipid measures are not good enough to predict cardiovascular outcomes in CKD patients [26,27]. The atherogenic lipid profile in CKD mostly defines the qualitative problems [28]. The impaired HDL configuration [29-32], increased serum levels of remnant lipoproteins, small very low-density and intermediate-density lipoproteins, and increased small and dense LDL cholesterol subclass (sdLDL) [28,33] are the features of atherogenic lipid profile in CKD. Compared to the larger LDL subclasses, sdLDL cholesterol is easily taken up by arterial tissue and is much more prone to the oxidation [34-37]. The elevated TG/HDL-C ratio reflects the presence of remnant lipoproteins and sdLDL levels [33,36], which play significant role in the increased cardiovascular risk [34,35].

The role of TG/HDL–C ratio as a marker of insulin resistance and a predictor for increased cardiovascular risk has been validated in several chronic metabolic disorders such as diabetes mellitus, hypertension, and nonalcoholic fatty liver disease [9-15,38]. According to our results elevated TG/HDL-C ratio is also significant cardiovascular risk marker for the CKD patients. TG/HDL-C ratio stands out as a significant predictor of cumulative mortality among many other well-established CVD risk factors such as age, blood pressure, HOMA-IR, calcium, phosphorous, PTH, uric acid and albumin. Regarding the high cardiovascular event rate, it is highly important to early recognize the CKD patients with higher risk and implement more intensive treatment modalities. Current biochemical markers to define the CVD risk are not technically perfect or widely available [39-41]. Furthermore, noninvasive tools of measuring endothelial dysfunction such as the FMD, carotid intima media thickness, or the ankle brachial index are time consuming or user dependent. To our knowledge, this is the first study to show that TG/HDL-C ratio is an independent determinant of endothelial dysfunction and a significant predictor for cardiovascular outcome. The median TG/HDL-C ratio established in this study is very similar to the previously reported cut off level of healthy people to predict the sdLDL phenotype [10].

This study may have several potential limitations. Although the TG/HDL-C ratio is a well-established correlate of insulin sensitivity, the HOMA-IR levels of the patients under and above the TG/HDL cut offs were not different in this study. It may be due to the fact that the HOMA index test is only a rough estimate of insulin sensitivity and may not be as accurate as clamp testing, or newer more accurate engines such as iHOMA-2 [42]. Owing to the observational nature of the study, some unknown or unincluded factors might have had an impact on cardiovascular endpoints. Also, the relatively small number of the patients and a substantial amount of subjects who were lost to follow up may be another drawback. Moreover, the incidence of CV events may be lower than expected, probably due to the fact that the CKD-1 and CKD-2 subjects were enrolled in the cohort. Finally, due to the exclusion of patients treated with ACEIs, ARBs or statins, the cohort may not be completely representative of the adult CKD population.

## Conclusions

The results of the present study show that the elevated TG/HDL-C ratio in CKD patients is a significant correlate of the well-established cardiovascular risk factors, a determinant of the endothelial dysfunction and a predictor of the increased cardiovascular mortality and morbidity. Being a simple, inexpensive, and reproducible marker of CVD risk, the TG/HDL-C ratio may be a novel and reliable predictor of CVD risk in subjects with CKD.

## References

[1] Go AS, Chertow GM, Fan D, McCulloch CE, Hsu CY (2004). Chronic kidney disease and the risks of death, cardiovascular events, and hospitalization. N Engl J Med.

[2] Stenvinkel P (2010). Chronic kidney disease: a public health priority and harbinger of premature cardiovascular disease. J Intern Med.

[3] Keith DS, Nichols GA, Gullion CM, Brown JB, Smith DH (2004). Longitudinal follow-up and outcomes among a population with chronic kidney disease in a large managed care organization. Arch Intern Med.

[4] Carrero JJ, Stenvinkel P (2010). Inflammation in end-stage renal disease—What have we learned in 10 years?. Semin Dial.

[5] Stenvinkel P, Carrero JJ, Axelsson J, Lindholm B, Heimbürger O, Massy Z (2008). Emerging biomarkers for evaluating cardiovascular risk in the chronic kidney disease patient: how do new pieces fit into the uremic puzzle?. Clin J Am Soc Nephrol.

[6] Levin NW, Handelman GJ, Coresh J, Port FK, Kaysen GA (2007). Reverse epidemiology: a confusing, confounding, and inaccurate term. Semin Dial.

[7] Rubin C, Nolin TD, Himmelfarb J (2007). Are biomarkers useful for assessing cardiovascular risk in patients with chronic kidney disease?. Curr Opin Nephrol.

[8] Hadaegh F, Hatami M, Tohidi M, Sarbakhsh P, Saadat N, Azizi F (2010). Lipid ratios and appropriate cut off values for prediction of diabetes: a cohort of Iranian men and women. Lipids Health Dis.

[9] Jeppesen J, Hein HO, Suadicani P, Gyntelberg F (1998). Triglyceride concentration and ischemic heart disease: an eight-year follow-up in the Copenhagen Male Study. Circulation.

[10] McLaughlin T, Reaven G, Abbasi F, Lamendola C, Saad M, Waters D (2005). Is there a simple way to identify insulin-resistant individuals at increased risk of cardiovascular disease?. Am J Cardiol.

[11] Gasevic D, Frohlich J, Mancini GB, Lear SA (2014). Clinical usefulness of lipid ratios to identify men and women with metabolic syndrome: a cross-sectional study. Lipids Health Dis.

[12] Vega GL, Barlow CE, Grundy SM, Leonard D, Defina LF (2014). Triglyceride-to-High-Density-Lipoprotein-Cholesterol Ratio Is an Index of Heart Disease Mortality and of Incidence of Type 2 Diabetes Mellitus in Men. J Investig Med.

[13] Onat A, Can G, Kaya H, Hergenç G (2010). “Atherogenic index of plasma” (log10 triglyceride/high-density lipoprotein-cholesterol) predicts high blood pressure, diabetes, and vascular events. J Clin Lipidol.

[14] Sung KC, Ryan MC, Kim BS, Cho YK, Kim BI, Reaven GM (2007). Relationships between estimates of adiposity, insulin resistance, and nonalcoholic fatty liver disease in a large group of nondiabetic Korean adults. Diabetes Care.

[15] Hermans MP, Ahn SA, Rousseau MF (2012). The atherogenic dyslipidemia ratio [log(TG)/HDL-C] is associated with residual vascular risk, beta-cell function loss and microangiopathy in type 2 diabetes females. Lipids Health Dis.

[16] National Kidney Foundation (2002). K/DOQI Clinical Practice Guidelines for Chronic Kidney Disease: Evaluation, Classification and Stratification. Am J Kidney Dis.

[17] Levey AS, Bosch JP, Lewis JB, Greene T, Rogers N, Roth D (1999). A more accurate method to estimate glomerular filtration rate from serum creatinine: a new prediction equation. Modification of Diet in Renal Disease Study Group. Ann Intern Med.

[18] Friedewald WT, Levy RI, Fredrickson DS (1972). Estimation of the concentration of low-density lipoprotein cholesterol in plasma, without use of the preparative ultracentrifuge. Clin Chem.

[19] Matthews DR, Hosker JP, Rudenski AS, Naylor BA, Treacher DF, Turner RC (1985). Homeostasis model assessment: insulin resistance and beta-cell function from fasting plasma glucose and insulin concentrations in man. Diabetologia.

[20] Chen BM, Xia LW, Zhao RQ (1997). Determination of N(G), N(G)-dimethylarginine in human plasma by high-performance liquid chromatography. J Chromatogr B Biomed Sci Appl.

[21] Haberka M, Mizia-Stec K, Gąsior Z, Mizia M, Janowska J, Holecki M (2009). Serum ADMA concentration – an independent factor determining FMD impairment in cardiac syndrome X. Ups J Med Sci.

[22] Celermajer DS, Sorensen KE, Gooch VM, Spiegelhalter DJ, Miller OI, Sullivan ID (1992). Non-invasive detection of endothelial dysfunction in children and adults at risk of atherosclerosis. Lancet.

[23] Corretti MC, Anderson TJ, Benjamin EJ, Celermajer D, Charbonneau F, Creager MA (2002). Guidelines for the ultrasound assessment of endothelial-dependent flow-mediated vasodilation of the brachial artery: a report of the International Brachial Artery Reactivity Task Force. J Am Coll Cardiol.

[24] Kanbay M, Siriopol D, Saglam M, Gulcan Kurt Y, Gok M, Cetinkaya H (2014). Serum sclerostin and adverse outcomes in non-dialyzed chronic kidney disease patients. J Clin Endocrinol Metab.

[25] Saab G, Bomback AS, McFarlane SI, Li S, Chen SC, McCullough PA (2012). The association of parathyroid hormone with ESRD and pre-ESRD mortality in the Kidney Early Evaluation Program. J Clin Endocrinol Metab.

[26] Tonelli M, Muntner P, Lloyd A, Manns B, Klarenbach S, Pannu N (2013). Association between LDL-C and risk of myocardial infarction in CKD. Alberta Kidney Disease Network. J Am Soc Nephrol.

[27] Holzmann MJ, Jungner I, Walldius G, Ivert T, Nordqvist T, Ostergren J (2012). Dyslipidemia is a strong predictor of myocardial infarction in subjects with chronic kidney disease. Ann Med.

[28] Quaschning T, Krane V, Metzger T, Wanner C (2001). Abnormalities in uremic lipoprotein metabolism and its impact on cardiovascular disease. Am J Kidney Dis.

[29] Yamamoto S, Yancey PG, Ikizler TA, Jerome WG, Kaseda R, Cox B (2012). Dysfunctional high-density lipoprotein in patients on chronic hemodialysis. J Am Coll Cardiol.

[30] Vaziri ND, Navab K, Gollapudi P, Moradi H, Pahl MV, Barton CH (2011). Salutary effects of hemodialysis on low-density lipoprotein proinflammatory and high-density lipoprotein anti-inflammatory properties in patient with end-stage renal disease. J Natl Med Assoc.

[31] Ansell BJ, Navab M, Hama S, Kamranpour N, Fonarow G, Hough G (2003). Inflammatory/antiinflammatory properties of high-density lipoprotein distinguish patients from control subjects better than high-density lipoprotein cholesterol levels and are favorably affected by simvastatin treatment. Circulation.

[32] Holzer M, Birner-Gruenberger R, Stojakovic T, El-Gamal D, Binder V, Wadsack C (2011). Uremia alters HDL composition and function. J Am Soc Nephrol.

[33] Rizzo M, Berneis K (2006). Low-density lipoprotein size and cardiovascular risk assessment. QJM.

[34] Varbo A, Benn M, Tybjaerg-Hansen A, Jorgensen AB, Frikke-Schmidt R, Nordestgaard BG (2013). Remnant cholesterol as a causal risk factor for ischemic heart disease. J Am Coll Cardiol.

[35] Hoogeveen RC, Gaubatz JW, Sun W, Dodge RC, Crosby JR, Jiang J (2014). Small Dense Low-Density Lipoprotein-Cholesterol Concentrations Predict Risk for Coronary Heart Disease: The Atherosclerosis Risk in Communities (ARIC) Study. Arterioscler Thromb Vasc Biol.

[36] Décary S, Dumon G, Lamarche B, Hoque JC, Tremblay AJ, Bergeron J (2010). Assessment of the validity of the frequently used lipid indices for predicting LDL peak particle diameter in a large cohort of 1955 normal and dyslipidemic subjects. Clin Brioche.

[37] Mikhailidis DP, Elisaf M, Rizzo M, Berneis K, Griffin B, Zambon A (2011). “European panel on low density lipoprotein (LDL) subclasses”: a statement on the pathophysiology, atherogenicity and clinical significance of LDL subclasses: executive summary. Curr Vasc Pharmacol.

[38] Després JP, Lemieux I, Dagenais GR, Cantin B, Lamarche B (2000). HDL-cholesterol as a marker of coronary heart disease risk: the Québec cardiovascular study. Atherosclerosis.

[39] Kielstein JT, Fliser D (2007). The past, presence and future of ADMA in nephrology. Nephrol Ther.

[40] Zoccali C, Yilmaz MI, Mallamaci F (2013). FGF23: a mature renal and cardiovascular risk factor?. Blood Purif.

[41] Yilmaz MI, Sonmez A, Ortiz A, Saglam M, Kilic S, Eyileten T (2011). Soluble TWEAK and PTX3 in nondialysis CKD patients: impact on endothelial dysfunction and cardiovascular outcomes. Clin J Am Soc Nephrol.

[42] Hill NR, Levy JC, Matthews DR (2013). Expansion of the homeostasis model assessment of β-cell function and insulin resista nce to enable clinical trial outcome modeling through the interactive adjustment of physiology and treatment effects: iHOMA2. Diabetes Care.

